# *KRAS* exon 2 codon 13 mutation is associated with a better prognosis than codon 12 mutation following lung metastasectomy in colorectal cancer

**DOI:** 10.18632/oncotarget.13697

**Published:** 2016-11-29

**Authors:** Stéphane Renaud, Francesco Guerrera, Joseph Seitlinger, Lorena Costardi, Mickaël Schaeffer, Benoit Romain, Claudio Mossetti, Anne Claire-Voegeli, Pier Luigi Filosso, Michèle Legrain, Enrico Ruffini, Pierre-Emmanuel Falcoz, Alberto Oliaro, Gilbert Massard

**Affiliations:** ^1^ Department of Thoracic Surgery, Strasbourg University Hospital, Strasbourg, France; ^2^ Department of Thoracic Surgery, Nancy University Hospital, Nancy, France; ^3^ Research Unit EA3430, Tumoral Progression and Micro-Environment, Epidemiological and Translational Approaches, Strasbourg University, Strasbourg, France; ^4^ Department of Thoracic Surgery, Azienda Ospedaliera Universitaria Città della Salute e della Scienza di Torino, Torino, Italy; ^5^ Department of Biostatistics, Strasbourg University Hospital, Strasbourg, France; ^6^ Department of General and Digestive Surgery, Strasbourg University Hospital, Strasbourg, France; ^7^ Department of Molecular Biology, Strasbourg University Hospital, Strasbourg, France

**Keywords:** lung metastasectomy, colorectal cancer, KRAS, surgery, codon

## Abstract

**Introduction:**

The utilization of molecular markers as routinely used biomarkers is steadily increasing. We aimed to evaluate the potential different prognostic values of *KRAS* exon 2 codons 12 and 13 after lung metastasectomy in colorectal cancer (CRC).

**Results:**

KRAS codon 12 mutations were observed in 116 patients (77%), whereas codon 13 mutations were observed in 34 patients (23%). KRAS codon 13 mutations were associated with both longer time to pulmonary recurrence (TTPR) (median TTPR: 78 months (95% CI: 50.61–82.56) vs 56 months (95% CI: 68.71–127.51), *P* = 0.008) and improved overall survival (OS) (median OS: 82 months vs 54 months (95% CI: 48.93–59.07), *P* = 0.009). Multivariate analysis confirmed that codon 13 mutations were associated with better outcomes (TTPR: HR: 0.40 (95% CI: 0.17–0.93), *P* = 0.033); OS: HR: 0.39 (95% CI: 0.14–1.07), *P* = 0.07). Otherwise, no significant difference in OS (*P* = 0.78) or TTPR (*P* = 0.72) based on the type of amino-acid substitutions was observed among KRAS codon 12 mutations.

**Materials and Methods:**

We retrospectively reviewed data from 525 patients who underwent a lung metastasectomy for CRC in two departments of thoracic surgery from 1998 to 2015 and focused on 150 patients that had *KRAS* exon 2 codon 12/13 mutations.

**Conclusions:**

**KRAS**

exon 2 codon 13 mutations, compared to codon 12 mutations, seem to be associated with better outcomes following lung metastasectomy in CRC. Prospective multicenter studies are necessary to fully understand the prognostic value of *KRAS* mutations in the lung metastases of CRC.

## INTRODUCTION

Colorectal cancer (CRC) remains one of the most diagnosed cancers and one of the leading causes of cancer-related deaths worldwide [1, 2]. Because the liver and lungs offer a favorable environment for CRC cells, up to 50% of the patients will experience metastases in these two locations [3]. Although still the subject of debate, lung metastasectomy is widely accepted by most surgical teams because, in selected populations, it leads to longer overall survival (OS) than conventional 5-fluorouracil (5-FU)-based chemotherapy regimens [4–6].

Several risk factors for poor outcomes have been identified to properly select patients who would clearly benefit from surgery, both in terms of OS and disease-free survival (DFS). Moreover, recent meta-analyses have helped to define main clinical prognostic factors [7]; in the past few years, there has been an increased understanding of the molecular alterations in cancer cells, with the identification of oncogenic drivers, suggesting that clinical factors could be a reflection of only gene mutations. These observations support the perspective of a molecular classification of patients, which carries with it the possibility of a better selection process for good surgical candidates [8–11].

In CRC, two proto-oncogenes have been extensively studied: *V-Ki-ras2 Kirsten rat sarcoma viral oncogene homolog* (*KRAS*) and *V-raf murine sarcoma viral oncogene homolog B1* (*BRAF*). The prognostic and predictive values of mutations of these two genes in metastatic CRC is now clearly defined, particularly leading to resistance to anti-Epidermal Growth Factor Receptor (EGFR) therapies [12]. On the other hand, although a growing number of publications have focused on evaluating the prognostic value of *KRAS* mutations after lung metastasectomy of CRC, clinical data are still inconsistent [8–11]. Furthermore, clinical studies on lung metastases of CRC seem to provide only a fleeting glimpse of what the extent the molecular biology of cancer cells could offer in our daily practice. Particularly, the tumoral heterogeneity of *KRAS* mutations remains intriguing. Indeed, recently published data have highlighted the existence of two distinct groups of *KRAS* cells: *KRAS*-dependent and *KRAS*-independent cells [13]. Moreover, it seems that according to *KRAS* amino-acid substitution, different downstream signaling pathways are activated [14], likely leading to different clinical behaviors such as different degrees of aggressiveness [15], different sites of metastasis [16] and/or different sensitivity to chemotherapy [17] and radiation therapy18. Hence, few studies on metastatic CRC tried to evaluate the prognostic significance of KRAS codon 12 and codon 13 mutations, with contradictory results [19–22]. However, to the best of our knowledge, this axis has not been yet investigated in lung metastases of CRC.

We thereby aimed to evaluate the different prognostic value of *KRAS* exon 2 codon 12 over codon 13 mutations in a large surgical cohort of resected lung metastases.

## RESULTS

According to the selection criteria, 150 patients with lung metastasis of CRC harboring *KRAS* mutations were included in this study. The clinical-pathological characteristics of these patients are displayed in Table 1. Median follow-up time was 56 months (IQR: 44).

**Table 1:** Demographic data and main covariates according to KRAS mutational status

	Total (*n*= 150)		Codon 12 (*n* = 116)		Codon 13 (*n* = 34)		*P*
	No.	%	No.	Col %	No.	Col %	
**Bevacizumab (Yes)**	37	25	27	23	10	29	0.465
**Age (Mean; SD)**	64	8	64	8	65	9	0.713
**Sex (male)**	83	55	67	58	16	47	0.328
**Median follow-up time (Median, IQR)**	56	44	55	43	58	47	0.89
CCI (*n **= 149)**							0.115
0	41	28	34	29	7	21	
1–2	56	38	38	33	18	55	
3–4	31	21	25	22	6	18	
>= 5	21	14	19	16	2	6	
**WHO-PS**							0.708
0	112	75	85	73	27	79	
1	34	23	27	23	7	21	
2	3	2	3	3			
3	1	1	1	1			
**Site of primary CRC**							0.489
Colon	74	49	59	51	15	44	
Rectum	76	51	57	49	19	56	
pT CRC (*n **= 148)**							0.61
I	16	11	12	11	4	12	
II	21	14	14	12	7	21	
III	88	59	69	61	19	56	
IV	23	16	19	17	4	12	
pN CRC (*n **= 148)**							0.007
0	87	59	64	56	23	68	
N1	31	21	29	25	2	6	
N2	28	19	21	18	7	21	
N3	2	1			2	6	
CEA (*n **= 143)**							0.584
0–5 ng/ml	94	66	71	65	23	70	
> 5 ng/ml	49	34	39	35	10	30	
**Chemotherapy**							0.062
None	39	26	29	25	10	29	
Peri-operative	27	18	16	14	11	32	
Post-operative	48	32	41	35	7	21	
Pre-operative	36	24	30	26	6	18	
**Side of metastasis**							
Bilateral	39	26	30	26	9	26	
Unilateral	111	74	86	74	25	74	
**DFS (> 12 months)**	121	81	95	82	26	76	0.481
**DFS (24 months)**	92	61	73	63	19	56	0.458
**Number of lung metastases (> 1)**	76	51	57	49	19	56	0.489
**Thoracic LNI (N+;** ***n*** **= 148)**	48	32	39	34	9	26	0.397
**Liver Metastasis**	24	16	19	16	5	15	0.815
**KRAS mutation subtype**							
G12A	7	5	7	6			
G12C	12	8	12	10			
G12D	49	33	49	42			
G12S	9	6	9	8			
G12V	39	26	39	34			
G13D	34	23			34	100	

* number of patients for whom the corresponding data were available. CCI: Charlson Comorbidity Index, WHO-PS: World Health Organization performance status, CRC: colorectal cancer, CEA: carcinoembryonic antigen, DFS: disease free survival, LNI: lymph node involvement, IQR: inter-quartile range.

Analyses of *KRAS* codon 12 transversions revealed 12 (8%) G12C, 39 (26%) G12V and 7 (5%) G12A. Analyses of *KRAS* codon 12 transitions revealed 49 (33%) G12D and 9 (6%) G12S. For KRAS codon 13, 34 (23%) cases of G13D transition were observed.

### Clinicopathological variables and *KRAS* mutations

Table 1 shows the distribution of clinicopathologic variables according to *KRAS* codon mutation.

### Survival analyses

**Figure 1:**
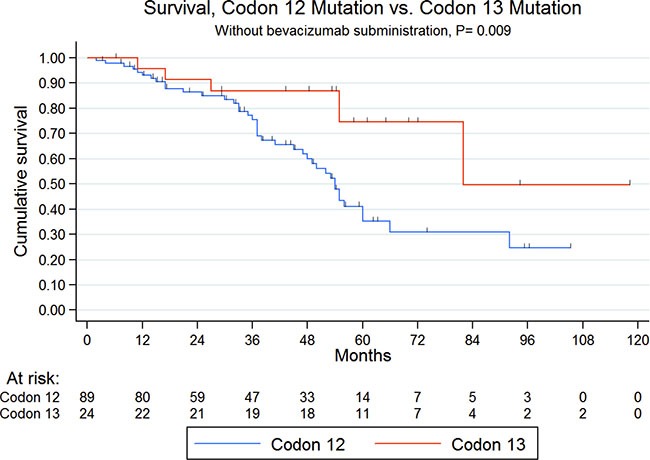
Kaplan-meier overall survival according to codon 12 or 13 mutations in patients not treated with bevacizumab

Ninety-five patients (63%) were reported to be alive, and 55 patients (37%) were dead during the follow-up period. Overall, the five-year survival rate was 42%. Survival analysis by *KRAS* codon mutation showed a non-significant difference between codon 12 and codon 13 mutations (median OS (mOS): 84 months vs 82 months, respectively; *P* = 0.167). However, bevacizumab showed a survival benefit when used in case of *KRAS* codon 12 mutations (mOS: Not reached (NR) vs 54 months, *P* < 0.001), but not in *KRAS* codon 13 mutations (mOS: NR vs 82 months; *P* = 0.48). Patients were then further analyzed with the exclusion of patients treated with bevacizumab. Hence, survival analysis in patients not treated with bevacizumab showed a significant difference between codon 12 and codon 13 mutations (mOS: 54 months vs 82 months, respectively; *P* = 0.009 - Figure 1). Otherwise, because among *KRAS* codon 12 mutations, G12D and G12V were the most frequent, other codon 12 mutations were pooled in the “other codon 12 mutations” group. There was no significant difference in mOS among *KRAS* codon 12 mutations (mOS: G12D 55, months (95% CI: 47.8–62) vs G12V, 55 months (95% CI 38.63–71.38) vs other codon 12 mutations, 53 months (95% CI 36.29–69.71); *P* = 0.78).

Because Food and Drug Administration approval of FOLFOX and bevacizumab in CRC were both obtained in 2004, we decided to compare OS between patients who underwent a lung metastasectomy before and after 2004. The median OS of patients included before 2004 (*n* = 100) was not significantly different from those included thereafter (*n* = 50) (median OS: 75 months (95% CI: 55.25–92.68) vs 83 months (95% CI: 72.32–102.18), respectively, *p* = 0.22)

Finally, in univariate analysis, male gender (*P* = 0.039), CCI (*P* < 0.001), WHO performance status *P* < 0.025), pT of CRC (*P* < 0.001), DFS (*P* = 0.006), thoracic LNI (*P* < 0.001) and liver metastasis *P* < 0.001) were found to have a negative effect on survival. Data are shown in Table 2.

**Table 2:** Uni- and multivariate analysis on overall survival (OS)

UNIVARIATE (*N* = 150)	Median survival (months)	*P*	[95% Conf. Interval]		MULTIVARIATE (without bevacizumab; *N*= 113)	Hazard Ratio	*P*	[95% Conf. Interval]	
**Mutation subtype**		0.167							
Codon 12	84		51	116					
Codon 13	82		–	–					
**Bevacizumab**		< 0.001							
No	56		54	82					
Yes	101		84	–					
**Mutation subtype without bevacizumab (*n*** **= 113)**		0.009			**Mutation subtype**				
Codon 12	54		47	60	Codon 12	1			
Codon 13	82		55	–	Codon 13	0.386	0.068	0.14	1.07
**Age**		0.112			**Age**				
< 60	92		92	–	< 60	1			
>= 60	82		55	101	>= 60	1.349	0.453	0.62	2.94
**Sex**		0.039			**Sex**				
Female	NR		84	–	Female	1			
Male	82		55	–	Male	1.195	0.645	0.56	2.55
**CCI**		< 0.001			CCI				
0	101		84	–	0	1			
1–2	NR		55	–	1–2	5.063	0.020	1.28	19.96
3–4	54		35	–	3–4	8.995	0.003	2.06	39.22
>= 5	17		17	–	>= 5	23.415	0.001	3.41	160.69
**WHO-PS**		0.025			**WHO-PS**				
0	92		66	–	0	1			
1	82		45	–	1	0.988	0.977	0.43	2.24
2	33		33	–	2	5.602	0.163	0.49	62.89
3	NR		–	–	3				
**Site of primary CRC**		0.819							
Colon	NR		55	–					
Rectum	84		60	101					
**pT Colon**		< 0.001			**pT Colon**				
T1	NR		52	–	T1	1			
T2	82		47	–	T2	4.691	0.075	0.85	25.79
T3	84		60	–	T3	1.857	0.406	0.43	7.99
T4	27		17	32	T4	9.190	0.029	1.25	67.49
**pN Colon**		0.066							
N0	82		55	–					
N1	60		33	84					
N2	NR		–	–					
N3	NR		–	–					
**CEA**		0.092			**CEA**				
0–5 ng/m	82		55	–	0–5 ng/m	0.895	0.779	0.41	1.94
> 5 ng/ml	92		84	–	> 5 ng/ml	1			
**Chemotherapy**		0.629							
None	82		55	–					
Peri-operative	NR		37	–					
Post-operative	92		55	–					
Pre-operative	60		37	84					
**Side of surgery**		0.401							
Unilateral	82		60	–					
Bilateral	101		38	–					
**Disease free survival**		0.004			**Disease free survival**				
< 24 months	101		82	–	< 24 months	1			
>= 24 months	56		53	92	>= 24 months	1.561	0.269	0.71	3.44
**Number of lung metastasis**		0.457							
1	101		60	–					
> 1	82		55	92					
**Thoracic LNI**		< 0.001			**Thoracic LNI**				
No	92		82	–	No	1			
Yes	50		37	56	Yes	2.075	0.104	0.86	5.01
**Liver Metastasis**		< 0.001			**Liver Metastasis**				
No	84		82	–	No	1			
Yes	47		37	–	Yes	1.509	0.379	0.61	3.77

CCI: Charlson Comorbidity Index, WHO: World Health Organization Performance Status, CRC: colorectal cancer, CEA: carcinoembryonic antigen, DFS: disease free survival, LNI: lymph node involvement.

Patients who had benefited from bevacizumab were excluded from the multivariate analysis to exclude the effect of bevacizumab on the OS of *KRAS* codon 12 patients. Hence, the multivariate-adjusted model showed that CCI and pT of primary CRC were independent negative predictors of survival. Finally, there was a trend toward improved OS in *KRAS* codon 13 mutations (HR = 0.386, *P* = 0.068). Data are displayed in Table 2.

### Time to pulmonary recurrence (TTPR)

**Figure 2:**
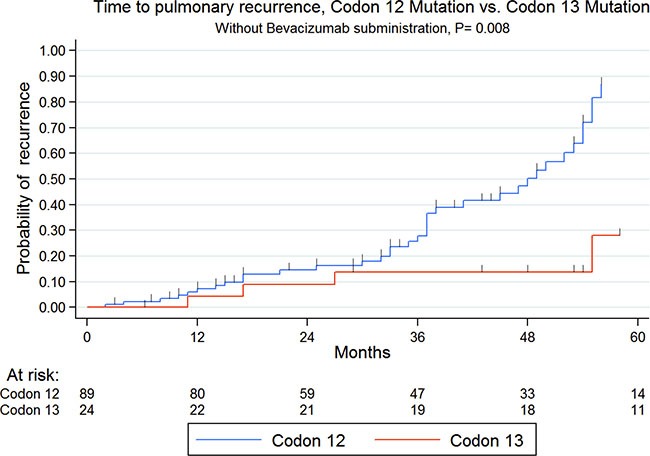
Kaplan-meier time to pulmonary recurrence according to codon 12 or 13 mutations in patients not treated with bevacizumab

One hundred forty patients (93%) were available for the TTPR analysis; 68 patients (48%) experienced a lung recurrence during the follow-up period. Overall, the 5-year TTPR rate was 45%. TTPR analysis by *KRAS* codon mutation showed a significant difference between codon 12 (Median TTPR (mTTPR): 60 months) and codon 13 mutations (mTTPR: NR; *P* = 0.041). However, bevacizumab showed a benefit on TTPR when used in the case of *KRAS* codon 12 mutations (mTTPR: 86 (95% CI: 57–115) vs 30 months (95% CI: 12–48), *P* < 0.001), but not in *KRAS* codon 13 mutations (mTTPR: 31 (95% CI: 15–47) vs 46 months (95% CI: 13–79.18), *P* = 0.39). Patients were then further analyzed, with the exclusion of patients treated with bevacizumab. Likewise, TTPR analysis in patients not treated with bevacizumab showed a significant difference between codon 12 (mTTPR: 51 months) and codon 13 mutations (mTTPR: NR; *P* = 0.008 - Figure 2). Otherwise, among *KRAS* codon 12 mutations, even shorter in case of G12V transversion (mTTPR: 21 months, 95% CI: 0–43), there was no significant difference with G12D transition (50 months, 95% CI: 42–58) and “other codon 12 mutations” (30 months, 95% CI: 0–68, *P* = 0.72).

Finally, in univariate analysis, the primary CRC site (*P* = 0.033), pT of CRC (*P* < 0.001), side of lung metastasis (*P* < 0.001), thoracic LNI (*P* = 0.015) and liver metastasis (*P* = 0.002) were found to have a negative effect on TTPR. Data are shown in Table 3.

**Table 3:** Uni- and multivariate analysis on time to pulmonary recurrence (TTPR)

UNIVARIATE (*N* = 140)	Median TTPR (months)	*P*	[95% Conf. Interval]		MULTIVARIATE (without bevacizumab; *N*= 113)	Hazard ratio	*P*	[95% Conf. Interval]	
**Mutation subtype**		0.041							
Codon 12	60		51	86					
Codon 13	NR		50	–					
**Bevacizumab**		0.049							
No	51		50	100					
Yes	92		70	98					
**Mutation subtype without bevacizumab (*n*** **= 113)**		0.009			**Mutation subtype without bevacizumab (*n*** **= 113)**				
Codon 12	51		41	60	Codon 12	1			
Codon 13	NR		50	–	Codon 13	0.40	0.033	0.17	0.93
**Age**		0.891							
< 60	86		50	–					
>= 60	60		51	98					
**Sex**		0.206							
Female	70		51	100					
Male	57		51	–					
**Site of primary CRC**		0.033			**Site of primary CRC**				
Colon	51		49	70	Colon	1.29	0.440	0.67	2.48
Rectum	86		60	98	Rectum	1			
**pT Colon**		< 0.001			**pT Colon**				
T1	98		51	112	T1	1			
T2	NR		20	–	T2	8.14	0.016	1.47	45.11
T3	60		51	86	T3	5.17	0.017	1.34	19.86
T4	15		12	24	T4	24.19	< 0.001	5.11	114.65
**pN Colon**		0.529							
N0	57		50	86					
N1	NR		16	–					
N2	NR		51	–					
N3	NR		–	–					
**CEA**		0.283							
0–5 ng/m	60		50	86					
> 5 ng/ml	92		51	100					
**Chemotherapy**		0.241							
None	100		50	–					
Peri-operative	57		40	–					
Post-operative	86		51	98					
Pre-operative	70		24	–					
**Side of surgery**		< 0.001			**Side of surgery**				
Unilateral	112		60	–	Unilateral	1			
Bilateral	40		16	57	Bilateral	5.65	< 0.001	2.62	12.21
**Disease free survival**		0.489							
< 24 months	60		51	98					
>= 24 months	70		51	112					
**Number of nodules**		0.409							
1	70		51	98					
> 1	60		50	–					
**Thoracic LNI**		0.015			**Thoracic LNI**				
No	86		60	92	No	1			
Yes	50		30	98	Yes	1.86	0.101	0.89	3.89
**Liver Metastasis**		0.002			**Liver Metastasis**				
No	86		57	92	No	1			
Yes	44		19	49	Yes	2.52	0.031	1.09	5.84

CCI: Charlson Comorbidity Index, WHO: World Health Organization Performance Status, CRC: colorectal cancer, CEA: carcinoembryonic antigen, DFS: disease free survival, LNI: lymph node involvement.

Patients who had benefited from bevacizumab were excluded from the multivariate analysis to exclude the effect of bevacizumab on the mTTPR of *KRAS* codon 12 patients. Hence, at multivariate analysis, pT of CRC, side of lung metastasis and liver metastasis demonstrated an independent negative effect on TTPR. Moreover, an independent effect on improved TTPR in *KRAS* codon 13 mutations was observed (HR 0.40, *P* = 0.033). Data are shown in Table 3.

## DISCUSSION

*In vitro* studies suggest a wide heterogeneity of *KRAS* mutations, suggesting different prognostic values of exon 2 codon 12 and codon 13 mutations. More specifically, exon 2 codon 12 mutations, in particular, G12D and G12V mutations, seem to be able to induce a more robust link with GTP molecules leading to higher resistance to GTPase activity compared to codon 13 mutations, in particular, G13D [23, 24]. On the other hand, it seems that *KRAS* codon 12 mutations, but not codon 13 mutations, are associated with a strong upregulation of vascular endothelial growth factor (VEGF) [24, 25], which is implicated in the promotion of lymphangiogenesis [26], which seems to be related to recurrence and decreased OS after lung metastasectomy in CRC [26]. These two observations suggest a higher aggressiveness of codon 12 mutations over codon 13 mutations. Although there have been no published data on the prognostic value of exon 2 codon mutations after lung metastasectomy in CRC to date, several authors have previously attempted to investigate its value in metastatic CRC. However, few data are available, and the studied populations have been heterogeneous, including all stages of CRC. In a collaborative study including 3439 CRC patients, Andreyev et al. [20] found a significant association between G12V mutation and both failure-free survival (HR: 1.3, *P* = 0.004) and OS (HR: 1.29, *P* = 0.008). However, the impact of this mutation was only observed in Duke's C stage, but not in Duke's B or in an advanced stage. In another work, Imamura et al. [21] confirmed in 1075 CRC patients the association between G12V mutation and worse OS (HR: 2.00 *P* = 0.0003), but without significant impact on prognosis of codon 13 mutations. In contrast, Samowitz et al. [22], in a large study of 1413 patients, although non-significant, found a 40% increase in short-term mortality from CRC in the case of codon 13 mutations. More recently, among 218 metastatic CRC patients, Dadduzio et al. [19] did not find any prognostic difference between codon 12 and codon 13 mutations. To the best of our knowledge, our study is the first to focus on the prognostic value of these 2 codon mutations after lung metastasectomy in CRC. Consistent with *in vitro* studies, our work suggests a higher aggressiveness of codon 12 over codon 13 mutations in terms of both OS and TTPR. Unlike previous authors [20, 21], we did not find any significant difference among codon 12 mutations. Indeed, even if G12V transversions were associated with decreased TTPR, there was no significant difference with all other types of codon 12 amino-acid substitutions. However, our population was small, and the absence of differences may have been related to a lack of power. Furthermore, the “other codon 12 mutations” group included different types of amino-acid substitutions; behaviors of each type of amino-acid substitution could not have been studied because of the small number of patients in each group. Indeed, *in vitro* studies have shown that the type of amino-acid substitution may activate different downstream signaling [14]. Hence, both *KRAS* G12C and G12V exhibited activated Ral signaling and decreased growth factor-dependent Akt activation, although the G12D mutation exhibited activated PI3K and MEK signaling. Consequently, it would not be surprising that different amino-acid substitutions confer different behavior to cancer cells after lung metastasectomy in CRC with different prognoses as observed in other cancers.

It is usually admitted that rectal cancer cells have a higher tropism to the lung than colon cancer cells. This might be partially explained by a mechanical theory, in which cancer cells can metastasize directly to the lung because of the drainage of the rectum by the rectal veins directly to the lower vena cava, meanwhile colon cancer cells must pass through the portal system and the liver, who plays the role of filter and stop cancer cells, before they can reach the lung. However, previous series have shown that CRC cells harboring *KRAS* mutations have a higher lung tropism than wild type or cancer cells harboring *BRAF* mutations [27]. This lung tropism of *KRAS* mutations may explain why we did not observe in our cohort a higher prevalence of rectal cancer.

As previously published by our team in a single-institutional study [28, 27], we confirmed in this multi-center study that bevacizumab may improve both OS and TTPR in patients with KRAS codon 12 mutations, but not with codon 13 mutations.

Finally, in our cohort known prognostic factors, namely thoracic LNI, liver metastases, DFS and pre-operative CEA did not significantly impact the OS. These observations may be related to our highly selected population, or on the other hand to the fact that mutational status is statistically more powerful to impact OS than other prognostic factors, and that these prognostic factors, as previously evoked, could be only reflections of mutational status [8].

However, our study must be interpreted with caution regarding a few limitations. First, it is a retrospective cohort study based on a relatively small sample size. Furthermore, 122 patients were from Strasbourg cohort, probably leading to a center effect. However, to the best of our knowledge, this is the largest published cohort on *KRAS* mutations in lung metastases of CRC, and a multicentre cohort seemed necessary to obtain enough statistical power in this highly selected population. Furthermore, our study covers a 17-year period during which there may have been changes in the management of patients; the different chemotherapy regimens used may have also influenced the survival of the study participants. However, a long study period was required to obtain enough statistical power. However, we did not observe a survival difference before and after FOLFOX and bevacizumab introduction. Furthermore, the molecular data were obtained from the primary CRC and not from the metastatic tumors. Consequently, there is remaining doubt regarding the degree of concordance between the primary and metastatic tumors, although Cejas et al. [29] reported a concordance rate of 94%. Finally, data on extra-thoracic recurrence were not available; this would be interesting to investigate in prospective studies.

In conclusion, to the best of our knowledge this is the first work on the largest published cohort of lung metastases with *KRAS* mutations showing that exon 2 codon 13 mutations harbor both better OS and TTPR compared to codon 12 mutations. This difference is supported by a molecular explanation because codon 12 mutations exhibit higher up-regulation of VEGF and more stable bonds between Ras and GTP. Our study adds more evidence to support that molecular biology may be helpful in our daily practice. To date, only a small portion of the large landscape of *KRAS* mutations have been explored. Prospective multicenter studies are necessary to understand the role of molecular biology in the proper selection of patients for lung metastasectomy of CRC. In particular, further studies are necessary to clarify the prognostic and predictive value of each *KRAS* amino-acid substitution.

## MATERIALS AND METHODS

This study was approved by the Ethics Committee of the French Society of Thoracic and Cardiovascular Surgeons (Approval Number: *2016–8-4–21–0-58-ReSt*). We retrospectively reviewed the data from 525 unselected and consecutive patients with metastatic CRC who underwent a lung metastasectomy in both the Thoracic Surgery Department of Strasbourg University Hospital (France) and Torino University Hospital (Italy) from January 1998 to December 2015. Patients for whom mutational status was unknown (i.e., *KRAS* and *BRAF*) (*n* = 92), who exhibited a *BRAF* mutation (*n* = 26), and who did not harbor *KRAS*/*BRAF* mutations (*n* = 257) were excluded from this study. All included patients were considered completely cured of their primary tumor at the time of the thoracic metastasectomy, and all pulmonary metastases were metachronous. In cases of extra-thoracic metastases, only patients with hepatic metastases were included. All thoracic resections were considered R0.

Pre-operative thoracic evaluation, lung metastasectomy procedures, covariates and data collection, and molecular analysis of *KRAS* mutations in codons 12 and 13 were performed as previously published [8].

### Covariates and data collection

Length of DFS was calculated from the surgery of the primary CRC to the first diagnosis of a thoracic or liver metastasis by imaging. OS was calculated from the first metastasectomy until death or the last follow-up. Time to pulmonary recurrence (TTPR) was defined as the time period between a thoracic metastasectomy and the first diagnosis of thoracic recurrence. Because patients were referred to our Departments of Thoracic Surgery by various oncologists from different centers, no uniform protocol for chemotherapy was performed. However, chemotherapy regimens generally consisted of fluoropyrimidines (5-fluorouracil (5-FU)), administered alone or in combination with oxaliplatin (Folfox/XelOx regimens) and/or irinotecan (Folfiri/Folfoxiri regimens). In some cases, bevacizumab was added at the discretion of the oncologist. The timing of chemotherapy was defined as follows: neo-adjuvant chemotherapy administered prior to thoracic surgery, adjuvant chemotherapy administered following lung metastasectomy, and POC was performed both in neo-adjuvant and adjuvant settings.

### Statistical analysis

Categorical data are presented as the number (percentage, %), and continuous data are presented as the mean with standard deviation (SD). Associations between Codon 12 and Codon 13 and clinical-pathological characteristics were assessed with the use of the Mann–Whitney U test for continuous variables and the chi-square test and Fisher's exact test for categorical variables as appropriate. The prognostic influence of predictors on OS and TTPR was assessed with a log-rank test and predictors associated with *P* ≤ 0.2 on univariate analysis were included in the multivariate models. A multivariate Cox proportional hazard model was employed to estimate the hazard ratios (HR) and 95% CI for the possible independent predictors of OS and TTR. All the tests were two-sided, and variables were considered significant for *P* values < 0.05. Statistical analyses were performed using Stata 13.1 (StataCorp LP, College Station, TX, USA).
